# Sonographic Retrobulbar Spot Sign in Diagnosis of Central Retinal Artery Occlusion: A Case Report

**DOI:** 10.21980/J8735P

**Published:** 2023-10-31

**Authors:** Emiliya Usheva, Dustin Williams, Haley Musgrave, Scott Zhou

**Affiliations:** * Corpus Christi Medical Center, Envision Physician Services, Corpus Christi, TX; ^ University of Texas, Southwestern Medical Center, Department of Emergency Medicine, Dallas, TX; † Baylor Scott & White Medical Center, Integrative Emergency Solutions, Irving, TX; ** University of Texas, Southwestern Medical Center, Department of Ophthalmology, Dallas, TX

## Abstract

Central retinal artery occlusion (CRAO) is a rare emergency department presentation with high morbidity and potential for long-term vision loss. Additionally, this finding requires an expeditious embolic workup for possible systemic pathology (i.e., stroke). The gold standard for diagnosis is visualization of a pale retina with a “cherry-red spot” on the fovea seen under dilated fundoscopic examination. However, performing a dilated fundoscopic exam is often not practical and technically challenging in the emergency room setting. Alternatively, point of care ultrasound is an inexpensive, non-invasive tool that is already highly utilized in the emergency department and can aid in diagnosis. In the case described in this report, a 66-year-old female presented to the emergency department with painless, monocular vision loss. Ultrasound showed a hyperechoic density on the distal aspect of the optic nerve (“retrobulbar spot sign”) and dilated fundoscopic exam showed right eye pale macula with cherry red spot, all consistent with CRAO. Here we present a case that suggests an opportunity for improvement in evaluation of monocular vision loss in the emergency department by adding bedside ocular ultrasound to aid in more rapid diagnosis of CRAO.

**Topics:**

Central retinal occlusion, vision loss, point-of-care ultrasound, ocular ultrasound, emboli.

[Fig f1-jetem-8-4-v5]

**Figure f1-jetem-8-4-v5:**
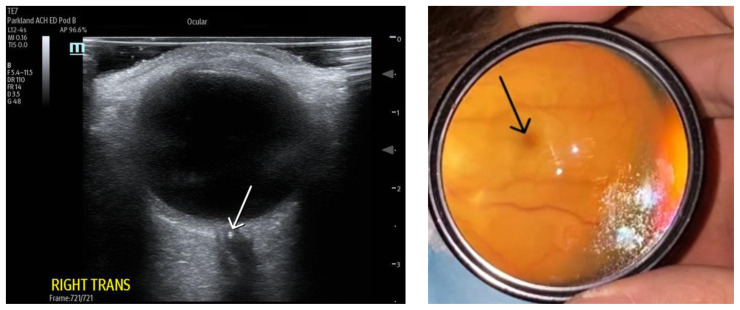
Ultrasound Video Link: https://youtu.be/miwLhjZ8WjA

## Brief introduction

The diagnosis of central retinal artery occlusion (CRAO) in the emergency department should prompt systemic evaluation for life threatening conditions (e.g. carotid occlusive or cardiac valve disease). If embolic etiology is suspected, an immediate referral to the nearest stroke center should be made as 1 in 4 patients with monocular vision loss demonstrate acute brain infarcts on diffusion-weighted imaging (DWI).^1^ Central retinal artery occlusion has similar pathophysiology and risk factors to ischemic stroke including cigarette smoking, hypertension, hyperlipidemia, diabetes, and cardiac disease. Arterio-arterial embolization is the most common cause of CRAO, with vasculitis accounting for just 4% of cases. While there is no proven treatment to reverse the vision loss caused by CRAO, its diagnosis is an important clinical indicator of possible embolic, inflammatory, or infectious disease and treatment is aimed at risk reduction and prevention of future adverse events.^2^ Furthermore, additional findings such as retrobulbar spot sign may be helpful in predicting the etiology of CRAO and thus aid in clinical approach and management decisions.^3^ Retrobulbar spot sign, a hyperechoic structure seen on trans-ocular ultrasound, is hypothesized to be the calcified portion of the embolus within the occluded retinal artery.^4^ Retrobulbar spot sign is highly specific for embolic CRAO and its presence excludes the diagnosis of temporal arteritis.^5^,^6^ In one study, detection of embolic CRAO using spot sign had a sensitivity of 83% and specificity of 100%.^5^,^6^ Thus, using bedside ultrasound to evaluate for spot sign can significantly expedite patient care.

## Presenting concerns and clinical findings

A 66-year-old female with no reported past medical history presents with one day of painless vision loss in her right eye. She awoke in the morning with blurry vision. Initially she could still see light and color out of her right eye but endorses severe blurriness, particularly in the infranasal visual field of the right eye, that has progressively worsened since onset. Patient reports a previous, brief episode of vision loss in the right eye 2 weeks prior that lasted ten minutes then resolved. No pain with eye movements. Denies any associated headaches or jaw claudication. No associated eye watering or redness.

Ophthalmological exam reveals gross orthotropia (normal eye alignment) with full ductions (normal uniocular rotations) and versions (synchronous simultaneous rotations of the two eyes in the same direction) without pain. Intraocular pressures normal on applanation, 13 and 15mmHg. Pupils noted to be round and reactive with no appreciable relative afferent pupillary defect. Patient noted to have significantly decreased visual acuity in her right eye and only able to count fingers at face in right eye (left eye visual acuity 20/25).

## Significant findings

On arrival, the ED team performed point-of-care ocular ultrasound, linear probe, 12 MegaHertz (MHz), that showed no evidence of retinal detachment, vitreous detachment, or vitreous hemorrhage. However, ultrasound did show a small, hyperechoic signal in the distal aspect of the optic nerve, concerning for embolus in the central retinal artery.

Ophthalmology was consulted in the ED for a dilated fundoscopic exam that was significant for a right eye pale macula with cherry red spot with a small area of edema supratemporal to disc, concerning for central retinal artery occlusion.

The bedside ocular ultrasound (B-scan) was significant for small, hyperechoic signal (white arrow) in the distal aspect of the optic nerve, concerning for embolus in the central retinal artery. Subsequent direct fundoscopic exam was significant for a pale macula with cherry red spot (black arrow), consistent with central retinal artery occlusion (CRAO).

## Patient course

Neurology was consulted for admission for rapid stroke-risk stratification. Advanced imaging with CT-angiography of the head and neck was significant for a narrowing of origin of the right internal carotid artery. The remainder of the stroke workup was unremarkable. The patient was started on dual antiplatelet therapy with aspirin and clopidogrel and a statin at time of discharge with minimal visual improvement on follow-up visit.

## Discussion

Central retinal artery occlusion is a rare ED presentation with high morbidity and potential for long-term vision loss secondary to retinal ischemia. The gold standard for diagnosis is visualization of a pale retina with a “cherry-red spot” on the fovea visualized under dilated fundoscopic examination. However, performing a dilated fundoscopic exam is often not practical and technically challenging in an emergency room setting. Point-of-care bedside ultrasound is an inexpensive, non-invasive tool that is already highly utilized in the emergency department to aid in diagnosis. This can be a rapid and useful modality for evaluation of many ocular complaints and can assist in rapid identification of a CRAO. Visualization of a hyperechoic density on the distal aspect of the optic nerve (“retrobulbar spot sign”) is ultrasonographic evidence of an embolic occlusion of the central retinal artery.

The central retinal artery is the first branch of the ophthalmic artery, which is the first branch of the internal carotid artery. There is a strong association between CRAO and carotid artery stenosis, and the most common cause of CRAO is arterio-arterial embolization of the central retinal artery from the ipsilateral internal carotid artery.^8^ The most important determinant of retinal damage and final visual outcome is the duration of occlusion of the central retinal artery.^8^ Similarly to acute ischemic stroke, intravenous thrombolytics may be considered in these patients with concern for embolic CRAO that present within 4.5 hours of onset of symptoms and without contraindications to thrombolytics.^8^ Thus, diagnosing CRAO requires an expeditious embolic workup for possible systemic pathology, as CRAO and cerebral ischemic stroke share much of the same underlying mechanisms and therapeutic approaches.

There are several additional conservative treatments suggested for the acute treatment of CRAO, however there are no clinical trials to support their usage and treatment is largely guided by consultation with specialists and may include: ocular massage, hyperbaric oxygen administration, anterior chamber paracentesis, reduction of intraocular pressure, and vasodilator medications. Thus, consultation and discussion with ophthalmology should be done as quickly as possible in order to expedite the multi-disciplinary care patients with CRAO require. With retrobulbar spot sign showing a sensitivity of 83% and specificity of 100% for CRAO, it can certainly aid in earlier consultation, definitive diagnosis, and treatment.^5^,^6^ Here we present a case that suggests an opportunity for improvement in evaluation of monocular vision loss in the emergency department by adding bedside ocular ultrasound to aid in more rapid diagnosis of CRAO.

## Supplementary Information







## References

[1] Helenius J Arsava EM Goldstein JN Cestari DM Buonanno FS Rosen BR Ay H 2012 Concurrent acute brain infarcts in patients with monocular visual loss Ann Neurol 72 286 293 10.1002/ana.23597 22926859 PMC3430972

[2] Flaxel Christina J Adelman Ron A Bailey Steven T Fawzi Amani Lim Jennifer I Atma Vemulakonda G Ying Gui-shuang Retinal and Ophthalmic Artery Occlusions Preferred Practice Pattern^®^ Ophthalmology 127 2 2020 P259 P287 0161-6420 10.1016/j.ophtha.2019.09.028 31757501

[3] Nedelmann M Graef M Weinand F Wassill KH Kaps M Lorenz B Tanislav C Retrobulbar Spot Sign Predicts Thrombolytic Treatment Effects and Etiology in Central Retinal Artery Occlusion Stroke 2015 Aug 46 8 2322 4 10.1161/STROKEAHA.115.009839 Epub 2015 Jun 25 26111890

[4] Altmann M Ertl M Helbig H Schömig B Bogdahn U Gamulescu M-A Schlachetzki F 2015 Low Endogenous Recanalization in Embolic Central Retinal Artery Occlusion—The Retrobulbar “Spot Sign” J Neuroimaging 25 251 256 10.1111/jon.12112 24641564

[5] Ertl M Altmann M Torka E Helbig H Bogdahn U Gamulescu A Schlachetzki F The retrobulbar “spot sign” as a discriminator between vasculitic and thrombo-embolic affections of the retinal blood supply Ultraschall Med 2012 Dec 33 7 E263 E267 10.1055/s-0032-1312925 Epub 2012 Sep 21 23023446

[6] Ertl M Altmann M Torka E Helbig H Bogdahn U Gamulescu A Schlachetzki F The retrobulbar spot sign in sudden blindness – sufficient to rule out vasculitis? Perspectives in Medicine 2012 Sep 1 1–12 408 413 10.1016/j.permed.2012.02.048

[7] Riccardi A Siniscalchi C Lerza R Embolic central retinal artery occlusion detected with point-of-care ultrasonography in the emergency department J Emerg Med 2016 50 4 e183 5 26879704 10.1016/j.jemermed.2015.12.022

[8] Mac Grory B Schrag M Biousse V Furie KL Gerhard-Herman M Lavin PJ Sobrin L Tjoumakaris SI Weyand CM Yaghi S American Heart Association Stroke Council; Council on Arteriosclerosis, Thrombosis and Vascular Biology; Council on Hypertension; and Council on Peripheral Vascular Disease Management of Central Retinal Artery Occlusion: A Scientific Statement From the American Heart Association Stroke 2021 Jun 52 6 e282 e294 10.1161/STR.0000000000000366 Epub 2021 Mar 8. Erratum in: Stroke. 2021 Jun;52(6):e309 33677974

