# Supplementary figures and images for: Sonographic Retrobulbar Spot Sign in Diagnosis of Central Retinal Artery Occlusion: A Case Report
Source: J Educ Teach Emerg Med. 2023 Oct 31;8(4):V5–8. doi: 10.21980/J8735P (PMC10631812; doi:10.21980/J8735P)

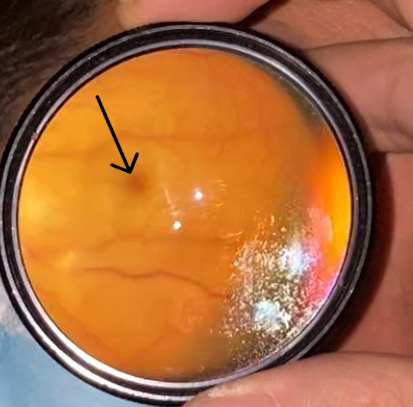

Supplement: Supplementary file 1 [file jetem-8-4-v5-supp1.jpg]

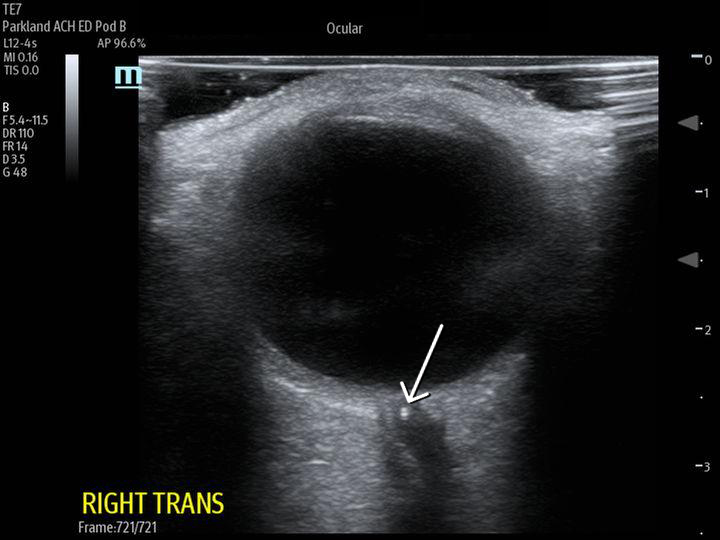

Supplement: Supplementary file 2 [file jetem-8-4-v5-supp2.jpg]
